# Integrated Transcriptome Analyses and Experimental Verifications of Mesenchymal-Associated TNFRSF1A as a Diagnostic and Prognostic Biomarker in Gliomas

**DOI:** 10.3389/fonc.2020.00250

**Published:** 2020-03-17

**Authors:** Biao Yang, Yuan-Bo Pan, Yan-Bin Ma, Sheng-Hua Chu

**Affiliations:** ^1^Department of Neurosurgery, Shanghai Ninth People's Hospital Affiliated to Shanghai Jiao Tong University School of Medicine, Shanghai, China; ^2^Department of Neurosurgery, Second Affiliated Hospital, School of Medicine, Zhejiang University, Hangzhou, China

**Keywords:** TNFRSF1A, subtype, prognosis, mesenchymal, proliferation, glioma

## Abstract

Gliomas are the most prevalent malignant primary brain tumors with poor outcome, and four different molecular subtypes (Mesenchymal, Proneural, Neural, and Classical) are popularly applied in scientific researches and clinics of gliomas. Public databases contain an abundant genome-wide resource to explore the potential biomarker and molecular mechanisms using the informatics analysis. The aim of this study was to discover the potential biomarker and investigate its effect in gliomas. Weighted gene co-expression network analysis (WGCNA) was used to construct the co-expression modules and explore the biomarker among the dataset CGGA mRNAseq_693 carrying 693 glioma samples. Functional annotations, ROC, correlation, survival, univariate, and multivariate Cox regression analyses were implemented to investigate the functional effect in gliomas, and molecular experiments *in vitro* were performed to study the biological effect on glioma pathogenesis. The brown module was found to be strongly related to WHO grade of gliomas, and KEGG pathway analysis demonstrated that TNFRSF1A was enriched in MAPK signaling pathway and TNF signaling pathway. Overexpressed TNFRSF1A was strongly related to clinical features such as WHO grade, and functioned as an independent poor prognostic predictor of glioma patients. Notably, TNFRSF1A was preferentially upregulated in the Mesenchymal subtype gliomas (Mesenchymal-associated). Knockdown of TNFRSF1A inhibited proliferation and migration of glioma cell lines *in vitro*. Our findings provide a further understanding of the progression of gliomas, and Mesenchymal-associated TNFRSF1A might be a promising target of diagnosis, therapy, and prognosis of gliomas.

## Introduction

Gliomas are the most prevalent malignant primary brain tumors (1). The origination of gliomas remains unclear, which are generally named according to the similar features to the normal glial cells (2). World Health Organization (WHO) gliomas are divided into low-grade gliomas (LGGs, grade I/II) and high-grade gliomas (HGGs, grade III/IV), respectively (3). Gliomas contain different Central Nervous System (CNS) cancers that generated from the glial cells such as astrocytomas, anaplastic astrocytomas, and glioblastoma multiformes (GBMs). Among these different gliomas, GBMs (grade IV) account for 70% gliomas and represent the malignant tumor of gliomas with a poor overall survival (OS) time of 12–18 months (4). In 2016, molecular characteristics, including isocitrate dehydrogenase (IDH) mutation and 1p/19q codeletion, are included into the revised the classification of the CNS Tumors (5). With the rapid advances in the sequencing technology, personalized molecular subtypes were constructed and more molecular markers are identified. Based on a study of The Cancer Genome Atlas (TCGA), glioblastoma multiforme (GBM) were divided into four molecular subtypes: Proneural, Neural, Classical and Mesenchymal by using by abnormalities in PDGFRA, IDH1, EGFR, and NF1 (6). Additionally, a study found that low vimentin was a favorable prognostic biomarker with a better response to temozolomide therapy, and vimentin expression was also related to grade of glioma patients (7). These molecular subtypes and biomarkers promote the diagnostic accuracy as well as diagnostic effect and prognosis assessment in glioma patients. Moreover, the researches on the molecular subtypes and markers remain a hot topic area of gliomas.

Weighted gene co-expression network analysis (WGCNA) is a systems bioinformatics method used to construct co-expression modules by the different correlation patterns among genes and select the hub genes related to the certain clinical feature by the intra-modular connectivity (IC) (8). WGCNA has already been successfully utilized in many studies (9, 10). Using the genome-transcriptiomic data of gastric cancer (GC) cell lines from Cancer Cell Line Encyclopedia (CCLE), Xiang et al. found that upregulated COL12A1 and LOXL2 were associated with IDO1 expression, and further biological experiments verified that IDO1 and COL12A1 could synergistically improve GC metastasis (11). Zhang et al. adopted WGCNA and protein-protein interaction (PPI) analysis to reveal that Tgfβ2, Wnt9a, and Fgfr4 were the hub genes regulating the differentiation of aging satellite cells (12). Thus, WGCNA is good at identifying the potential biomarker and its mechanisms of diseases.

Tumor necrosis factor α (TNF-α) is a highly active cytokine participating in the signaling pathway of necrosis or apoptosis (13). TNF receptor (TNFR1) regulates cell survival, apoptosis and inflammation via activating TNF-induced NF-κB signaling pathway (14). TNF receptor superfamily member 1A (TNFRSF1A) is a member of the TNF receptor superfamily of proteins (15). TNFRSF1A polymorphism rs4149584 was found to alleviate the severity of multiple sclerosis patients by decreasing age at disease onset and retarding disease progression (16). TNFRSF1A also functions as a target gene of miR-29a promoting the apoptosis of AR42J cells in acute pancreatitis (14). A recent study found that a four-gene panel of CD44, ABCC3, TNFRSF1A, and MGMT could act as the prediction of GBM patients' therapy response (17). However, the role of TNFRSF1A in the development of gliomas still remains unclear.

In this study, TNFRSF1A was identified as a biomarker of molecular subtypes and an independent prognostic factor of gliomas based on the integrated bioinformatics analyses. TNFRSF1A is highly expressed in glioma tissues compared with normal brain tissues, and is related to poor prognosis of glioma patients. Knockdown of TNFRSF1A inhibited glioma cell proliferation and migration. These findings show that TNFRSF1A might be a significantly independent prognostic factor and a potential therapeutic target of gliomas.

## Materials and Methods

### Downloading and Preprocessing of mRNA Data and Clinical Information

The glioma expression profiles with clinical information were downloaded from the Gene Expression Omnibus (GEO; http://www.ncbi.nlm.nih.gov/geo/) database, including GSE4271, GSE4290, GSE4412, GSE13041, and GSE68848. The GSE4271 and GSE4412 datasets were originated from GPL96, and the dataset GSE13041 with 267 samples included three platforms GPL96, GPL570, and GPL8300. And the dataset GSE4271 was used for correlation analyses between TNFRSF1A expression and microvascular proliferation or necrosis. And the two datasets GSE4290 and GSE68848 were generated from same microarray platform GPL570. In addition, RNA-seq data of LGGs and GBM were downloaded from The Cancer Genome Atlas (TCGA; http://cancergenome.nih.gov/). Moreover, three datasets with mRNA data and clinical information (containing molecular subtypes, WHO grade, IDH mutation, 1p/19q codeletion, and chemotherapy and radiotherapy status), including mRNA-array_301 with 301 glioma samples, mRNAseq_325 with 325 gliomas and mRNAseq_693 with 693 gliomas, were acquired from the Chinese Glioma Genome Altas (CGGA; http://www.cgga.org.cn/) database. Data preprocessing in different datasets were implemented. And all of these datasets were used to conduct correlation analyses between TNFRSF1A expression and clinical features (age, molecular subtypes, WHO grade and so on). Moreover, the datasets (mRNA-array_301, mRNAseq_325 and mRNAseq_693 of CGGA, GSE4271, GSE4412, GSE68848, TCGA_glioma, and TCGA_LGG) were used to perform survival and Cox regression analyses.

As we have previously described Molnar et al. (13), differentially expressed genes (DEGs) were identified between 504 HGG samples and 188 LGG samples in the dataset CGGA mRNAseq_693 using package Limma in R 3.5.0. And the expression profile of DEGs in CGGA mRNAseq_693 was used for WGCNA.

Additionally, Oncomine (https://www.oncomine.org) is an online database containing numerous available microarray data of the different tumors. Comparison statistical analysis of TNFRSF1A expression between brain/ CNS cancers and corresponding normal samples with the threshold of *P* < 0.05 and fold-change (FC)>0 was implemented using this tool (14). Gene Expression Profiling Interactive Analysis 2 (GEPIA2; http://gepia.cancer-pku.cn/index.html), based on the both TCGA and GTEx databases, is used to perform the correlation and survival analyses in this study (15). The Human Protein Atlas (HPA; https://www.proteinatlas.org), an immunohistochemistry-based expression data of the all proteins in normal and tumor samples, was used to determine the expression level of the protein TNFRSF1A in this study (16).

### Construction of WGCNA

On the basis of the profile CGGA mRNAseq_693 carrying gene, weighted gene co-expression network was constructed using the WGCNA package in R (17). After cutting off the outliers, a proper soft-thresholding power (β) was determined to satisfy the scale-free topology, and then the adjacencies were turned into topological overlap matrix (TOM). The correlations between co-expression modules and clinical data (including age, subtypes, WHO grades and so on) were calculated by Pearson's correlation coefficient to explore the potentially biological value of the modules. Top 10 mRNAs with the highest IC in the interested module were selected as hub genes for the next analyses.

### Functional Annotation for the Brown Module

For all mRNAs in the interested brown module, the Database for Annotation, Visualization and Integrated Discovery (DAVID, https://david.ncifcrf.gov/) v6.8 was used for functional enrichment analyses along with Gene Ontology (GO) and Kyoto Encyclopedia of Genes and Genomes (KEGG) pathway analyses (18, 19). The functional analysis results were displayed using the GOplot package in R (20). *P* < 0.01 was set as the cutoff criteria.

### Cell Culture and RNA Interference

The human GBM cell lines (U87 and U251) were provided by Department of Neurosurgery, Shanghai Ninth People's Hospital Affiliated to Shanghai Jiao Tong University School of Medicine, and were cultured in Dulbecco's modified Eagle's medium (DMEM) supplemented with 10% fetal bovine serum (FBS) at 37°C in a 5% CO_2_ atmosphere. And small interfering RNAs siRNAs specifically targeting TNFRSF1A (siRNA1, siRNA2, siRNA3), siRNA scrambled control (si-NC) were obtained from RiboBio (Guangzhou, China). qRT-PCR and western blotting was used to evaluate the specificity and efficacy of siRNAs and scrambled control.

### RNA Extraction and Quantitative Real-Time Polymerase Chain Reaction (qRT-PCR)

In brief, total RNA was collected from cell lines using TRIzol reagent (Invitrogen, Carlsbad, CA, USA) for the reverse transcription reaction. GAPDH was used as an internal control. The primer sequences used were listed as below: TNFRSF1A, forward: 5′-TGCCATGCAGGTTTCTTTCT-3′, reverse: 5′-CACAACTTCGTGCACTCCAG-3′; GAPDH, forward: 5′-AGGTCGGAGTCAACGGATTT-3′, reverse: 5′-ATCTCGCTCCTGGAAGATGG-3′. The gene expression level was normalized to the internal control GAPDH. The relative expression level of TNFRSF1A was determined using the 2–ΔΔCt.

### Western Blotting

As we have previously described Chen et al. (21), total protein samples were then separated by 15% SDS-PAGE and transferred onto PVDF membranes. Membranes were incubated overnight at 4°C after blocking with primary antibody anti-TNFRSF1A. Following washing and incubation, membranes were incubated with secondary antibody in TBST. Brands were detected using an enhanced chemiluminescence system.

### Cell Counting Kit-8 (CCK-8) Assay

To explore cell proliferation ability, cells were cultured in 96-well plates with a density of 5 × 103 cells/well. CCK8 was performed according to the manufacture's instruction. After 4 h of incubation at 37°C in the presence of 5% CO_2_, the absorbance of each well was measured at 450 nm by using a spectrophotometer at day 1, 2, 3, 4, 5, and 6 after initial plating.

### Colony Formation Assay

According to the manufacture's instruction cells were inoculated and cultivated onto the 6-well plates (1 × 103 cells/well). The cells were then fixed with 4% paraformaldehyde for 30 min and stained with crystal violet for 30 min at room temperature. Final results were shown as an average of three independent assays.

### Transwell Assay

Cell migration ability was conducted using Transwell assay. A total of 105 cells in FBS-free DMEM were added to the top chamber, while DMEM containing 10% FBS was added to the bottom chamber. After 24 h of incubation, the migrated cells were fixed with 100% methanol and stained with 0.5% crystal violet at room temperature. Images captured with a digital camera. The values for migration and invasion were obtained by counting three randomly selected fields.

### Statistical Analyses

Statistical analyses were calculated by using SPSS 21.0 (San Diego, CA, USA), GraphPad Prism 7.0 (San Diego, CA, USA) or software R 3.5.0. The Pearson's correlation coefficient analysis was used to analyze the correlations. Comparisons of two groups were performed by unpaired student's *t*-test. Receiver operating characteristic (ROC) curves, and Cox regression analyses were implemented using R 3.5.0. Kaplane-Meier survival analysis was performed by GEPIA2 or R 3.5.0. Experimental data are displayed as the mean ± the standard deviation (SD), and *P* < 0.05 was considered to be statistically significant.

## Results

### Construction of WGCNA

**Figure 1:**
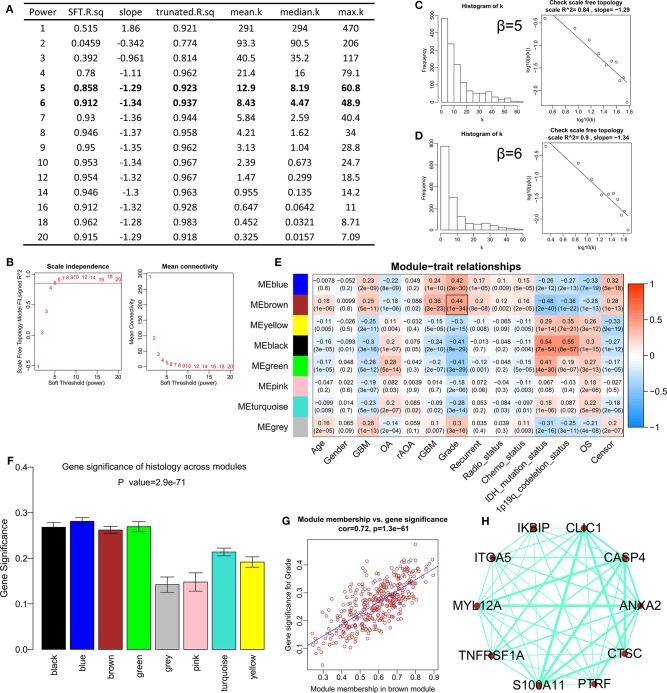
Construction of the weighted gene co-expression network analysis in CGGA mRNAseq_693. **(A)** Table of network topology for various soft-thresholding powers (β). **(B)** Analyses of network topology for various soft-thresholding powers, and the scale-free topology were set as 0.85 roughly. **(C)** Histogram of connectivity distribution and checking the scale free topology (*R*^2^ = 0.84) when β = 5. **(D)** Histogram of connectivity distribution and checking the scale-free topology (*R*^2^ = 0.9) when β = 6. **(E)** Module-trait relationships. The brown module was strongly associated with the WHO grade. **(F)** Distribution of average gene significance and errors in different modules. **(G)** Scatter plots for correlations between module membership and gene significance in brown module. **(H)** The network for top 10 hub genes in brown module.

After difference analysis, a total of 1456 DEGs were identified between HGGs and LGGs with the cut-off criteria of *P* < 0.05 and |log2FC|>0.5 using R language. Next, the expression profile of these DEGs was used to build the co-expression expression network using the WGCNA package in R. No outlier was identified (Supplementary Figure 1). The network topology was shown when different soft-thresholding powers (β) were set, and when the power was 5, the topology was roughly calculated being more than 0.85 (Figures 1A,B). Connectivity distribution and the scale-free topology were further analyzed. In fact, the scale-free topology (*R*^2^) was 0.84 when β = 5 (Figure 1C), and on the other hand *R*^2^ = 0.9 when β = 6 (Figure 1D). Clustering dendrogram of genes was shown in Supplementary Figure 2, and atotal of seven modules and a gray module were screened (Figure 1E). From the module-trait relationships in Figure 1E, the brown module consisted of 378 genes was found to be the most significant module related to WHO grade (*r* = 0.44, *P* = 1e−34). The gene significances of the modules were shown in Figure 1F, and the correlation (correlation = 0.72, *P* = 1.3e−61) was analyzed between module membership and gene significance of the brown module (Figure 1G). Top 10 genes with highest intra-modular connectivity was extracted as hub genes in brown module, including CLIC1, ANXA2, CASP4, ITGA5, MYL12A, S100A11, TNFRSF1A, CTSC, PTRF and IKBIP (Figure 1H).

### Functional Enrichment Annotations of the Brown Module

**Figure 2:**
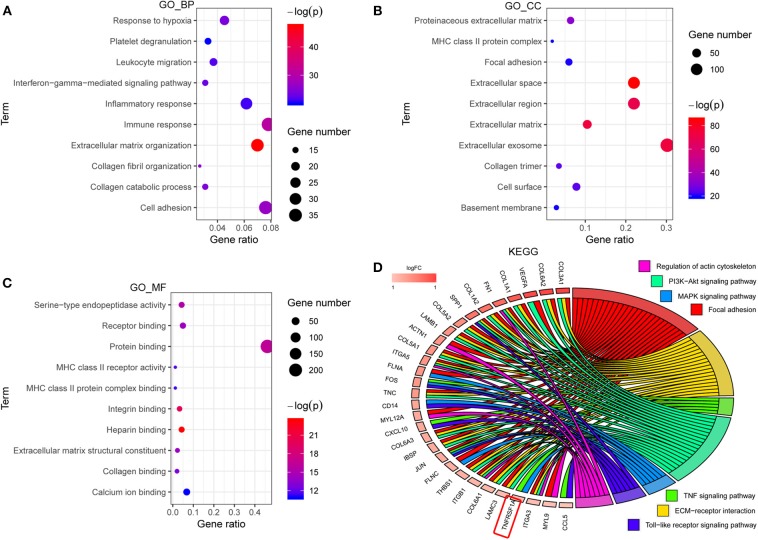
The functional enrichment analyses of all genes in brown module. **(A–C)** The plots for the top 10 significant enrichment annotations of all genes in brown module for BP, CC and MF in GO, respectively. **(D)** KEGG analysis was visualized using the package GOplot in R. The plot included 7 KEGG pathway terms. The genes with at least two terms, and the term which includes at least five genes are shown in this plot. Log_2_foldchange (logFC) represents the difference between high-grade gliomas and low-grade gliomas in the dataset CGGA mRNAseq_693. GO, Gene Ontology; BP, biological process; CC, cellular component; MF, molecular function; KEGG, Kyoto Encyclopedia of genes and genomes.

GO analyses contained biological process (BP), molecular function (MF) and cell component (CC), and top 10 significant terms in each category were chosen using the online database DAVID in this study. The GO analysis results showed that all genes of the brown modules were significantly associated with extracellular matrix organization and immune response in BP (Figure 2A); extracellular space and extracellular exosome in CC (Figure 2B); and heparin binding and integrin binding in MF (Figure 2C). For the KEGG analysis, the genes with at least two terms, and the term which includes at least five genes are shown in this plot. Ultimately, seven signaling pathway terms were extracted, and TNFRSF1A was enriched in MAPK signaling pathway (Figure 2D).

### TNFRSF1A Was Overexpressed and Related to Clinical Features in Gliomas

**Figure 3:**
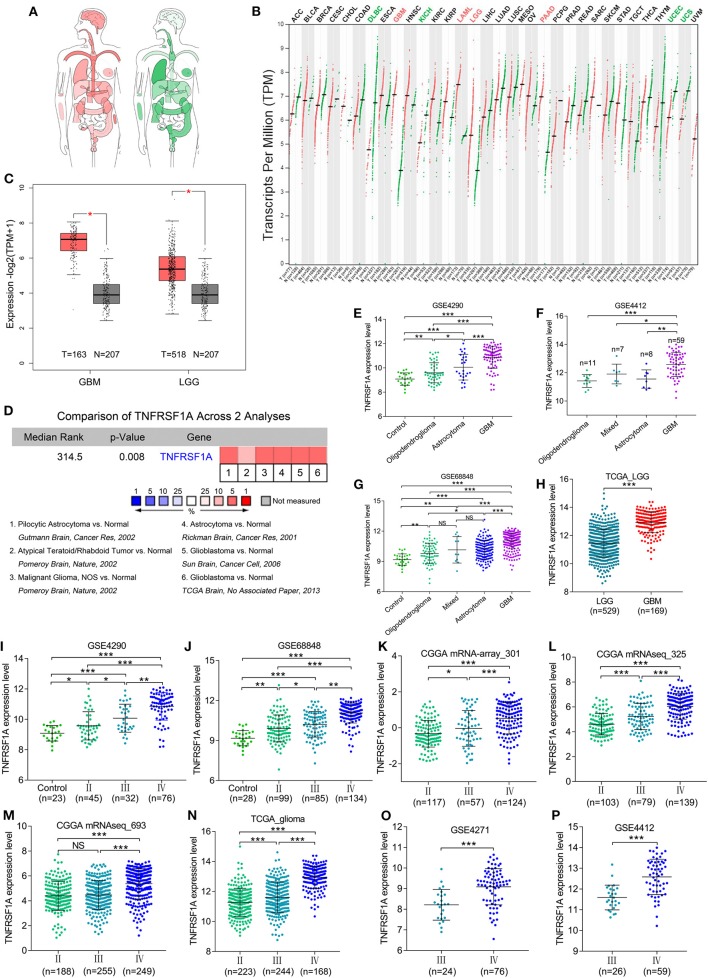
The expression levels of TNFRSF1A in gliomas. **(A)** Bodymap for the median expression of TNFRSF1A in tumor (red) and normal (green) samples using GEPIA 2. **(B)** The TNFRSF1A expression profile across all tumor samples and paired normal tissues using GEPIA2. The red plot represents the tumor sample and the green one indicates the normal sample. The TNFRSF1A expression in tumor samples is significantly upregulated compared the corresponding normal tissues when the name of the tumor is red (*P* < 0.05); on the contrary, the green indicates that TNFRSF1A expression in the tumor samples is downregulated (*P* < 0.05). **(C)** TNFRSF1A expression in GBM and LGG samples was upregulated compared with the normal brain samples based on the TCGA normal and GTEx data from the online tool GEPIA2 (*P* < 0.05). **(D)** The comparison analyses across 6 datasets from the Oncomine database showed that TNFRSF1A in gliomas was significantly upregulated compared to normal samples with the cut-off of *P* < 0.05 and foldchange>2 as well as gene rank = 10%. **(E–H)** TNFRSF1A expression was analyzed in various histologies of gliomas. **(I–P)** The expression of TNFRSF1A is positively associated with WHO grades in the eight datasets, including GSE4290, GSE68848, CGGA (mRNA-array_301, mRNAseq_325 and mRNAseq_693), TCGA_glioma, GSE4271 and GSE4412, respectively. *P < 0.05; **P < 0.01; ***P < 0.001.

**Figure 4:**
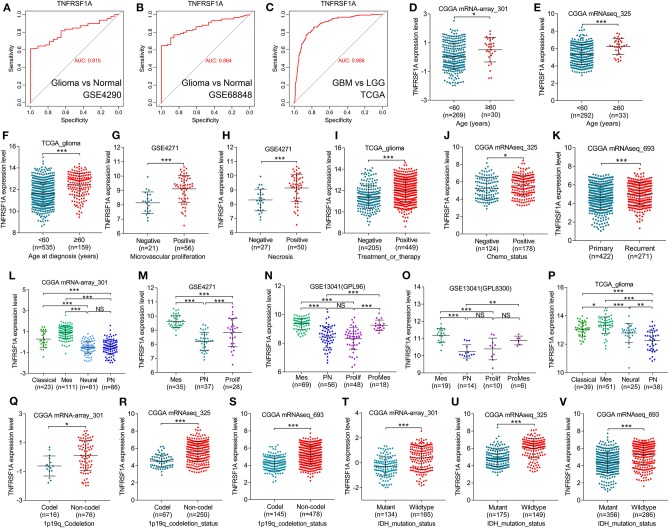
TNFRSF1A expression was associated with clinical features and was a subtype-associated molecular biomarker in glioma patients. **(A–C)** ROC analysis found that TNFRSF1A was able to predict the pathological diagnosis of gliomas in GSE4290, GSE68848 and TCGA, respectively (AUC>0.7). **(D–J)** The relationships between TNFRSF1A expression and clinical features (including ages, microvascular proliferation, necrosis, chemotherapy, and therapy) were analyzed. **(K)** The expression of TNFRSF1A in recurrent gliomas was upregulated compared with primary samples in CGGA mRNAseq_693. **(L–P)** TNFRSF1A expression in different subtypes of gliomas was analyzed in the five datasets, including CGGA mRNA-array_301, GSE4271, GSE13041 (GPL96), GSE13041 (GPL8300), and TCGA_glioma, respectively. **(Q–V)** The associations between TNFRSF1A expression and molecular characteristics (1p/19q codeletion and IDH mutation) were determined in the datasets CGGA mRNA-array_301, mRNAseq_325 and mRNAseq_693, respectively. Mes, mesenchymal; Prolif, proliferative; PN, proneural; ProMes, proliferative and mesenchymal; Codel, 1p/19q codeletion; ROC, receiver operating characteristic; AUC, area under the curve. *P < 0.05; **P < 0.01; ***P < 0.001.

KEGG result showed that MAPK signaling pathway, which played an important role in the development of gliomas, contained 13 genes of this study. Additionally, TNFRSF1A was identified by merging of top 10 hub genes in brown module and these 13 genes of MAPK signaling pathway (Supplementary Figure 3). From the bodymap from the online tool GEPIA2 in Figure 3A, change fold of the median expression of TNFRSF1A between the brain tumor (red) and the corresponding normal brain (green) samples was obvious, which suggested that TNFRSF1A expression might have a significant difference between brain tumors and normal brain tissues. Furthermore, the TNFRSF1A expression prolife of various tumors from the GEPIA2 illustrated that TNFRSF1A was significantly overexpressed in four kinds of tumors compared with the corresponding normal samples, including GBM, LAML (acute myeloid leukemia), LGG and PAAD (pancreatic adenocarcinoma) (Figure 3B). The boxplots precisely revealed that TNFRSF1A expression was upregulated in gliomas (LGGs and GBMs) compared with the normal corresponding samples using the GEPIA2 (Figure 3C). Moreover, the comparison analyses across 6 datasets from the Oncomine database showed that TNFRSF1A in gliomas was significantly upregulated compared with normal samples with the cut-off of *P* < 0.05 and FC > 2 as well as gene rank = 10% (Figure 3D). On the other hand, TNFRSF1A expression increased in the order of control (normal brain) tissues, oligodendroglioma, astrocytoma, and GBM (Figures 3E–G). Interestingly, TNFRSF1A expression was upregulated in the order of control samples, LGGs and HGGs (Figures 3H–J). More importantly, the expression level of TNFRSF1A was positively associated with WHO grade in the eight datasets, including GSE4290, GSE68848, CGGA (mRNA-array_301, mRNAseq_325 and mRNAseq_693), TCGA_glioma, GSE4271 and GSE4412, respectively (Figures 3I–P). The clinical correlation analyses also showed that TNFRSF1A expression was significantly associated with histology (Supplementary Tables 1–3, 5, 6) and WHO grade (Supplementary Tables 1–6) of gliomas. Furthermore, ROC analysis found that TNFRSF1A was capable of predicting the pathological diagnosis of gliomas in GSE4290, GSE68848 and TCGA, respectively (AUC>0.7) (Figures 4A–C). Collectively, TNFRSF1A expression was upregulated in gliomas than normal samples, and related to glioma histology and WHO grade.

Besides, boxplots and clinical correlation analyses showed that TNFRSF1A expression was significantly associated with other clinical features, including age, microvascular proliferation, necrosis, chemotherapy and radiotherapy status, recurrence and KPS. Glioma samples with older age (≥60) or microvascular proliferation or necrosis or Chemo_status (chemotherapy status) or treatment and therapy status have higher TNFRSF1A expression compared with the corresponding samples (Figures 4D–J). As shown in Figure 4K, recurrent gliomas have higher expression level of TNFRSF1A than primary gliomas. The clinical correlation analyses of the six datasets showed that TNFRSF1A expression was significantly associated with age (Supplementary Tables 1–3, 5, 6), gender (Supplementary Tables 4, 5), microvascular proliferation (Supplementary Table 4), necrosis (Supplementary Table 4), chemo_status (Supplementary Table 3), Radio_status (radiotherapy status) (Supplementary Table 1), treatment and therapy status (Supplementary Tables 5, 6) and PRS_type (primary/ recurrent/ secondary) (Supplementary Table 3) of human patients with gliomas, and its expression had no significance with some clinical features such as gender (Supplementary Tables 1–3, 6), race (Supplementary Tables 5, 6), chemo_status (Supplementary Tables 1, 2), Radio_status (Supplementary Tables 2, 3), KPS (Karnofsky performance score) (Supplementary Tables 5, 6). Taken together, TNFRSF1A expression was upregulated in gliomas, increased as the advances of WHO grade, and was related to various clinical features (including age, microvascular proliferation, necrosis, chemotherapy and radiotherapy status, recurrence and KPS) in gliomas, suggesting that TNFRSF1A might be a potential target gene related to diagnosis, treatment and prognosis of gliomas.

### TNFRSF1A Was a Novel Mesenchymal-Associated Biomarker in Molecular Classification of Gliomas

TCGA group classified HGGs into four subtypes: Classical, Mesenchymal, Neural and Proneural based on the molecular biomarkers such as 1p/19q codeletion and IDH mutation. Based on the datasets from GEO, TCGA and CGGA, TNFRSF1A could differentiate the various subtypes of gliomas, and more importantly, its expression level in Mesenchymal subtype gliomas was higher than other subtypes (Figures 4L–P; Supplementary Tables 1, 4, 6). Likewise, TNFRSF1A expression of gliomas carrying 1p/19q codeletion or IDH mutation was lower than the corresponding glioma samples (Figures 4Q–V; Supplementary Tables 1–3). Therefore, TNFRSF1A was a novel Mesenchymal-associated biomarker in molecular classification of gliomas.

### TNFRSF1A Was an Independent Prognostic Indicator of OS in Gliomas

**Figure 5:**
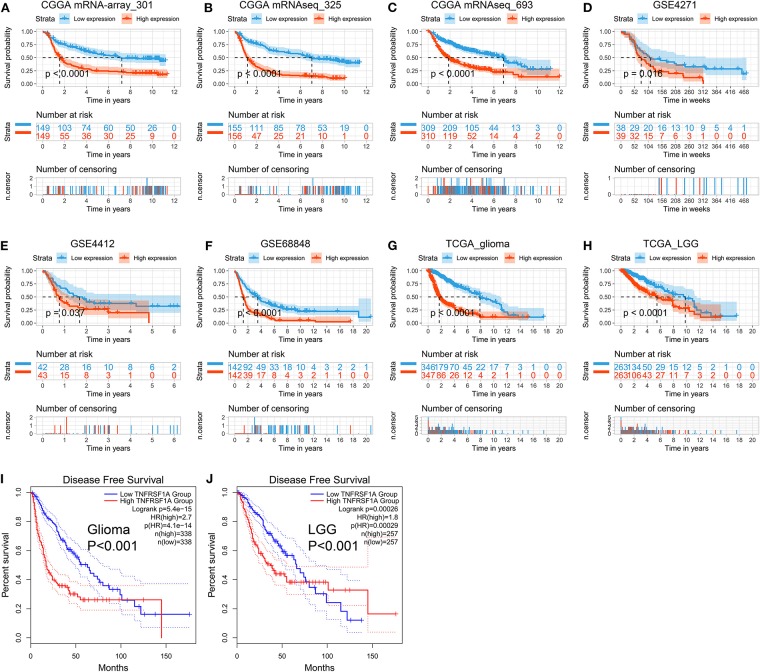
TNFRSF1A expression is associated with poor prognosis of glioma patients. **(A–H)** The Kaplan-Meier plots of TNFRSF1A in the eight datasets, including CGGA (mRNA-array_301, mRNAseq_325, and mRNAseq_693), GSE4271, GSE4412, GSE68848, TCGA_glioma, and TCGA_LGG, respectively. **(I,J)** The glioma patients with high TNFRSF1A expression have shorter overall survival (OS) and disease-free survival (DFS) compared the low TNFRSF1A group using the GEPIA2. Red line indicates the high TNFRSF1A expression group and blue line represents the low TNFRSF1A expression group.

The Kaplan-Meier plots were performed to examine the relationship between the TNFRSF1A expression and OS of glioma patients. Prior to the analysis, glioma samples without survival information were cut off and obtained samples were classified into two groups: low expression group and high expression group, according to the median of TNFRSF1A expression. For the dataset CGGA mRNA-array_301, the low TNFRSF1A expression group (*n* = 149) had a favor prognosis of OS than the high expression (*n* = 149) (Figure 5A). Likewise, the high TNFRSF1A expression group (*n* = 156, 310, 39, 43, 142, 347, and 263 respectively) had a poor prognosis compared with the corresponding low group (*n* = 155, 309, 38, 42, 142, 346, and 263 respectively) in the other seven datasets, including CGGA (mRNAseq_325 and mRNAseq_693), GSE4271, GSE4412, GSE68848, TCGA_glioma, and TCGA_LGG, respectively (Figures 5B–H). Furthermore, glioma or LGG patients with high TNFRSF1A expression had a shorter disease-free survival (DFS) than the low expression group using GEPIA2 (Figures 5I,J). Thus, TNFRSF1A expression was associated with poor prognosis in gliomas.

Besides, whether TNFRSF1A expression was an independent prognostic indicator of OS was determined using the univariate and multivariate Cox regression analyses. In the dataset CGGA mRNA-array_301, univariate Cox regression analysis result revealed that a total of 10 factors were associated with OS of gliomas, including TNFRSF1A expression, age, WHO grade, PRS_type, histology, TCGA_subtypes, Radio_status, Chemo_status, IDH_mutation_status and 1p19q_codeletion_status (Table 1). However, multivariate analysis result illustrated that five factors were identified as independent prognostic indicators, including TNFRSF1A expression (*P* = 0.007, HR = 1.90, 95% CI = 1.19–3.01), WHO grade (*P* = 0.048, HR = 1.50, 95% CI = 1.00–2.25), TCGA_subtypes (*P* = 0.048, HR = 1.56, 95% CI = 1.00–2.42), Radio_status (*P* = 0.011, HR = 0.36, 95% CI = 0.16–0.79) and 1p19q_codeletion_status (*P* = 0.029, HR = 0.24, 95% CI = 0.07–0.87) (Table 1). Similarly in the another dataset CGGA mRNAseq_325, univariate analysis revealed that a total of 9 factors were associated with OS of gliomas, including TNFRSF1A expression, age, WHO grade, PRS_type, histology, Radio_status, Chemo_status, IDH_mutation_status and 1p19q_codeletion_status (Table 2). Multivariate result illustrated that six factors were identified as independent prognostic indicators, including TNFRSF1A expression (*P* = 0.049, HR = 1.16, 95% CI = 1.00–1.34), age (*P* = 0.013, HR = 1.02, 95% CI = 1.00–1.03), WHO grade (*P* < 0.001, HR = 2.04, 95% CI = 1.59–2.63), PRS_type (*P* =0.013, HR = 1.99, 95% CI = 1.16–3.42), Chemo_status (*P* = 0.036, HR = 0.69, 95% CI = 0.49–0.98), and 1p19q_codeletion_status (*P* < 0.001, HR = 0.31, 95% CI = 0.18–0.55) (Table 2). Taken together, these results from the two datasets revealed that TNFRSF1A expression and WHO grade as well as 1p19q_codeletion_status were independent prognostic indicators of OS in gliomas.

**Table 1:** Cox regression analysis of TNFRSF1A expression as a survival indicator of gliomas in CGGA mRNA-array_301.

**Parameter**	**Univariate analysis**			**Multivariate analysis**		
	***P***	**HR**	**95%CI**	***P***	**HR**	**95%CI**
Age (≥60 vs. <60 years)	***P* < 0.001**	1.04	1.03–1.05	0.475	1.01	0.98–1.05
Gender (Male vs. Female)	0.164	1.24	0.92–1.67	NA	NA	NA
WHO grade (II vs. III vs. IV)	***P* < 0.001**	2.70	2.23–3.26	**0.048**	1.50	1.00–2.25
PRS_type (Primary vs. Recurrent vs. Secondary)	***P* < 0.001**	2.22	1.67–2.94	0.313	1.63	0.63–4.23
Histology (A vs. AA vs. AO vs. AOA vs. GBM vs. O vs. OA vs. rA vs. rAA vs. rAO vs. rAOA vs. rGBM vs. sGBM)	***P* < 0.001**	1.14	1.08–1.19	0.627	1.05	0.87–1.27
TCGA_subtypes (Classical vs. Mesenchymal vs. Neural vs. Proneural)	***P* < 0.001**	0.62	0.53–0.72	**0.048**	1.56	1.00–2.42
Radio_status (Positive vs. Negative)	**0.012**	0.58	0.37–0.89	**0.011**	0.36	0.16–0.79
Chemo_status (Positive vs. Negative)	**0.021**	1.43	1.05–1.94	0.425	0.76	0.39–1.49
IDH_mutation_status (Mutant vs. Wildtype)	***P* < 0.001**	0.38	0.28–0.52	0.639	0.82	0.35–1.90
1p19q_codeletion_status (Codel vs. Non-codel)	***P* < 0.001**	0.12	0.04–0.40	**0.029**	0.24	0.07–0.87
TNFRSF1A expression (High vs. low)	***P* < 0.001**	1.64	1.39–1.94	**0.007**	1.90	1.19–3.01

*CGGA, the Chinese Glioma Genome Altas; WHO, World Health Organization; A, astrocytomas; AA, anaplastic astrocytomas; AO, anaplastic oligodendrogliomas; AOA, anaplastic oligoastrocytomas; GBM, glioblastoma multiforme; O, oligodendrogliomas; OA, oligoastrocytomas; rA, recurrent astrocytomas; rAA, recurrent anaplastic astrocytomas; rAO, recurrent anaplastic oligodendrogliomas; rAOA, recurrent anaplastic oligoastrocytomas; rGBM, recurrent glioblastoma multiforme; sGBM, secondary glioblastoma multiforme; TCGA, The Cancer Genome Atlas; NA, not analyze. The bold values is aimed to emphasize the parameter with P < 0.05*.

**Table 2:** Cox regression analysis of TNFRSF1A expression as a survival indicator of gliomas in CGGA mRNAseq_325.

**Parameter**	**Univariate analysis**			**Multivariate analysis**		
	***P***	**HR**	**95%CI**	***P***	**HR**	**95%CI**
Age (≥60 vs. <60 years)	***P* < 0.001**	1.03	1.02–1.04	**0.013**	1.02	1.00–1.03
Gender (Male vs. Female)	0.613	0.93	0.71–1.23	NA	NA	NA
WHO grade (II vs. III vs. IV)	***P* < 0.001**	2.74	2.28–3.30	***P* < 0.001**	2.04	1.59–2.63
PRS_type (Primary vs. Recurrent vs. Secondary)	***P* < 0.001**	2.12	1.75–2.57	**0.013**	1.99	1.16–3.42
Histology (A vs. AA vs. AO vs. AOA vs. GBM vs. O vs. OA vs. rA vs. rAA vs. rAO vs. rAOA vs. rGBM vs. rOA vs. sGBM)	***P* < 0.001**	1.12	1.08–1.16	0.559	0.97	0.88–1.07
Radio_status (Positive vs. Negative)	***P* < 0.001**	0.52	0.36–0.74	0.167	0.75	0.50–1.13
Chemo_status (Positive vs. Negative)	**0.004**	1.55	1.15–2.08	**0.036**	0.69	0.49–0.98
IDH_mutation_status (Mutant vs. Wildtype)	***P* < 0.001**	0.38	0.29–0.51	0.989	0.10	0.67–1.49
1p19q_codeletion_status (Codel vs. Non-codel)	***P* < 0.001**	0.17	0.10–0.28	***P* < 0.001**	0.31	0.18–0.55
TNFRSF1A expression (High vs. low)	***P* < 0.001**	1.60	1.43–1.79	**0.049**	1.16	1.00–1.34

*CGGA, the Chinese Glioma Genome Altas; WHO, World Health Organization; A, astrocytomas; AA, anaplastic astrocytomas; AO, anaplastic oligodendrogliomas; AOA, anaplastic oligoastrocytomas; GBM, glioblastoma multiforme; O, oligodendrogliomas; OA, oligoastrocytomas; rA, recurrent astrocytomas; rAA, recurrent anaplastic astrocytomas; rAO, recurrent anaplastic oligodendrogliomas; rAOA, recurrent anaplastic oligoastrocytomas; rGBM, recurrent glioblastoma multiforme; rOA, recurrent oligoastrocytomas; sGBM, secondary glioblastoma multiforme; NA, not analyze. The bold values is aimed to emphasize the parameter with P < 0.05*.

### Protein Expression of TNFRSF1A in Glioma Tissues Was Upregulated Compared to Normal Brain Tissues by IHC Staining From the HPA

**Figure 6:**
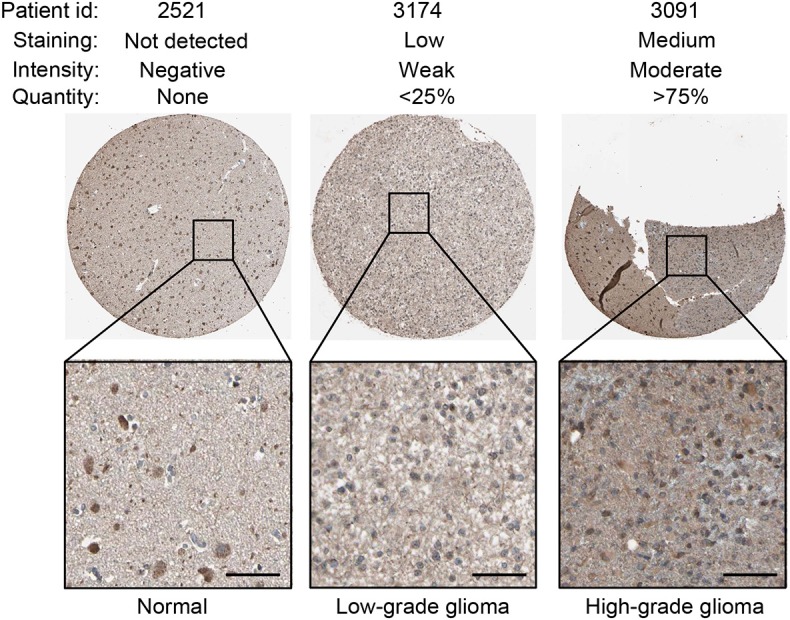
IHC staining showed that TNFRSF1A expression increased in the order of normal brain tissue, low-grade glioma and high-grade glioma. Antibody TNFRSF1A was HPA004102, and representative TNFRSF1A staining images were shown. Scale bar, 50 μm.

Using the same antibody HPA004102 from the IHC-based HPA database, protein expression level of GNG5 protein increased in the order of normal brain tissue, LGG and HGG among different patients (Figure 6), which further confirmed the important role of TNFRSF1A in gliomas at the protein level.

### Downregulation of TNFRSF1A Inhibited Glioma Cell Proliferation and Migration *in vitro*

**Figure 7:**
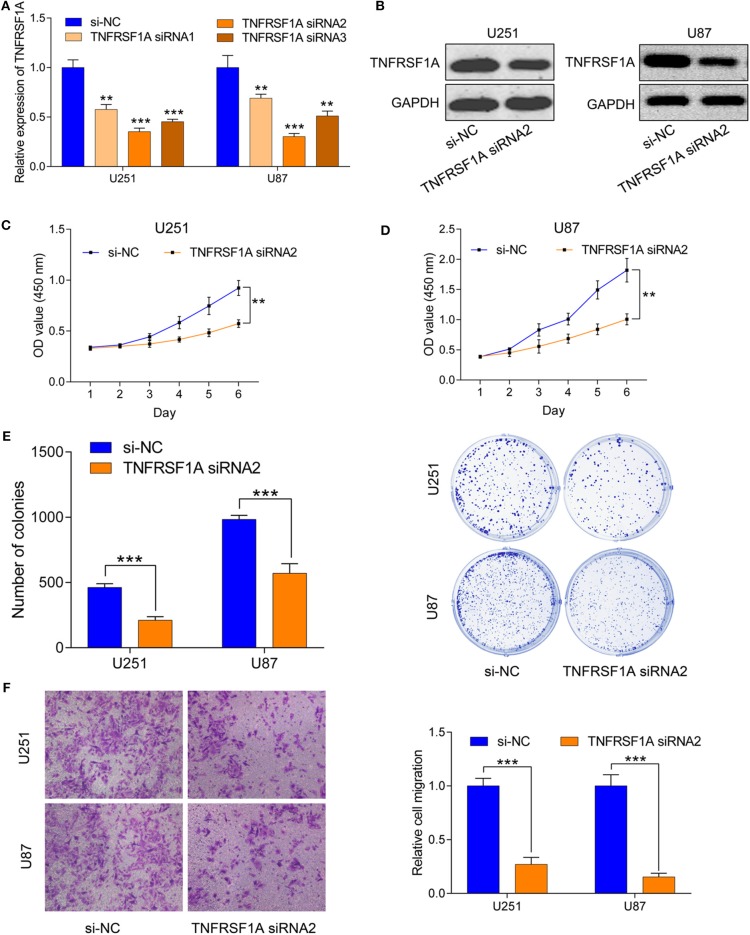
Downregulation of TNFRSF1A inhibited glioma cell proliferation and migration *in vitro*. **(A)** Efficiency of siRNAs at the mRNA level was validated in U251 and U87 cells by qRT-PCR. And siRNA2 has highest efficiency and si-NC was used as control. **(B)** Expression levels of the protein TNFRSF1A in glioma cells transfected with siRNA2 or si-NC were examined by western blotting. **(C,D)** Effects of TNFRSF1A knockdown on glioma development were determined by Cell Counting Kit-8 assay in U251 and U87, respectively. **(E)** Proliferative capacity of TNFRSF1A knockdown in in U251 and U87 cells transfected with siRNA2 or si-NC by using colony formation assay. **(F)** Transwell migration assay was performed in U87 and U251 transfected with si-NC or TNFRSF1A siRNA2. Representative images of migration cells are displayed. The number of migration cells was counted and was inversely related to the expression levels of TNFRSF1A. **P < 0.01; ***P < 0.001.

To investigate the role of TNFRSF1A in glioma, TNFRSF1A was knocked down in U251 and U87. After transfection of glioma cells with si-NC or TNFRSF1A siRNAs, knockdown efficiency of TNFRSF1A siRNA2 was most obvious in both U251 and U87 (Figure 7A). Furthermore, TNFRSF1A expression was dramatically reduced at the protein level while TNFRSF1A was knocked down using western blotting analysis (Figure 7B). Based on the CCK-8 assay, proliferative capacity of glioma cells transfected with TNFRSF1A siRNA2was significantly compared to those treated with si-NC (Figures 7C,D). The results of colony formation assay further demonstrated that colony numbers of glioma cells were significantly inhibited while TNFRSF1A was silencing (Figure 7E). As shown in Figure 7F, transwell assay showed that TNFRSF1A knockdown significantly reduced the migration of glioma cells. Taken together, downregulation of TNFRSF1A inhibited glioma cell proliferation and migration *in vitro*.

## Discussion

Gliomas are the most prevalent malignant primary brain tumors with poor survival prognosis (1), and according to abnormalities of several biomarkers GBMs are subgrouped into four molecular subtypes: Classical, Mesenchymal, Neural and Proneural, by the TCGA team (6). Owing to therapeutic resistance and high recurrent rate of gliomas, individual treatment based on the molecular biomarkers has gained more attentions (18). Recently, more transcriptome databases, such as TCGA, GEO and CGGA, have been constructed to be free to the public and provide abundant gene expression profiles and clinical information. In this study, an integrated bioinformatics analysis of the public databases found that the brown co-expression module and the biomarker TNFRSF1A was identified to be strongly related to WHO grade of gliomas. Further informatics analyses revealed that upregulated TNFRSF1A was significantly associated with clinical features, and functioned as an independent prognostic indicator of OS. Its effect and molecular mechanisms in glioma cell lines were verified using the biological experiments *in vitro*.

WGCNA, a system informatics method, is used to construct the network with scale-free distribution and is good at identifying the potential biomarker and its mechanisms of diseases. To date, there are also some similar researches on gliomas. For example, Xu et al. used WGCNA to screen four genes (OSMR, SOX21, MED10, and PTPRN) in the yellow module related to survival time and applied the Cox proportional hazards (PH) regression model to assess their prognostic significance (19). Likewise, Liang et al. identified a GBM-related module and adopted the Cox PH regression model to extract six prognostic lncRNAs like LINC00641 and LBX2-AS1 (20). A recent study have reported that an OS-associated module was identified and a lncRNA was screened by survival analyses based on the CGGA with 88 samples carrying compete clinical information (21). The three studies above adopted WGCNA to a co-expression module which related to survival time (or overall survival) or GBM, but there some limitations. One problem is that single survival time without status (including dead or alive) could not represent the exact survival time of tumor patients, and the ages at the diagnosis is the another factor related to the real survival time. Secondly, although GBM (glioma WHO IV) is the most serious pathology type of gliomas histologically, GBMs were classified into four subtypes (Classical, Mesenchymal, Neural and Proneural) (6) and different subtypes have different biological behaviors such as survival time from weeks to years (22). Also, when the module was selected based on the GBM diagnosis, there are only two events (Positive or Negative), which could not take full advantage of WGCNA because construction of co-expression modules depends on the patterns of the parameter changes. And in this study, we adopted WHO grade (I, II, III, and IV) of gliomas to investigate the co-expression modules. To a certain extent, this strategy could improve the risk stratification of different glioma patients according to the severity of gliomas and make best of WGCNA. Notably, the result of this study showed that the brown module was strongly related to WHO grade (*r* = 0.44, *P* = 1e-34), and no module was found to be associated with OS or GBM.

Moreover, glioma samples with some features (including older ages, high WHO grades, microvascular proliferation and necrosis) had a higher expression level of TNFRSF1A than the corresponding samples, suggesting that TNFRSF1A might be associated with poor prognosis. Furthermore, consistent with the above results, survival analyses and Cox regression analyses demonstrated that human patients with gliomas carrying high TNFRSF1A expression had a shorter OS or DFS than the low expression patients, and TNFRSF1A functioned as an independent prognostic indicator of OS. Thus, TNFRSF1A might be a novel biomarker of diagnosis, therapy, and prognosis related to progression of gliomas.

Besides, TNFRSF1A was verified to be associated to the molecular classification of gliomas, and it was highly upregulated in Mes subtype and downregualted in PN subtype of gliomas in this study. Compared with the translational histological classification, molecular classification highly took individual difference into account and added molecular characteristics to this classification. And glioma patients with molecular characteristics such as IDH mutation or 1p/19q codeletion had a lower expression level of TNFRSF1A than the glioma samples without these characteristics. From the above results that TNFRSF1A expression was an independent prognostic indicator related to poor prognosis in gliomas, therefore, gliomas with IDH mutation or 1p/19q codeletion had a longer survival time, which was consistent with the conclusions of the previous studies. A study with 941 malignant glioma samples found that the PN and Neural, and Classical and Mes subtypes frequently occurred in LGGs and HGGs, respectively (23). On the other hand, survival analyses demonstrated that the Mes subtype gliomas behaved a poor survival outcome and conversely the PN subtype gliomas displayed a better prognosis (23). Some previous studies found that IDH mutation was associated with 1p/19q codeletion (24), and glioma patients with IDH mutation had a longer median overall survival time than samples with IDH wildtype (25). Collectively, TNFRSF1A expression was significantly associated with the molecular characteristics and subtypes, and its overexpression was related to a poor prognosis, indicating that TNFRSF1A was a novel and promising Mesenchymal-associated biomarker in molecular subtypes of gliomas and might play critical roles in target therapy in the future.

KEGG enrichment analysis showed that TNFRSF1A could regulate the tumorignenesis of gliomas via activating the MAPK signaling pathway. Knockdown of TNFRSF1A suppressed glioma cells proliferation, migration and invasion *in vitro*, which further confirmed the important values of TNFRSF1A on glioma progression.

Overall, TNFRSF1A was identified by an integrated informatics analyses such as WGCNA, and transcriptome analyses further revealed that TNFRSF1A expression was upregulated in glioma samples compared with the normal brain samples. Moreover, the expression level of TNFRSF1A was associated with WHO grade and other clinical features such as molecular subtypes, and was found to serve as an independent prognostic indicator of OS in gliomas. Knockdown of TNFRSF1A suppressed the progression of glioma cell lines *in vitro*. These finding suggested that TNFRSF1A might be a promising biomarker of diagnosis, therapy and prognosis in Mesenchymal subtype gliomas.

## Data Availability Statement

The datasets for this study can be found in the GEO (http://www.ncbi.nlm.nih.gov/geo/), TCGA (http://cancergenome.nih.gov/), and CGGA (http://www.cgga.org.cn/).

## Author Contributions

S-HC and BY conceived and designed the study. BY performed the analysis procedures. Y-BP and Y-BM analyzed the results and contributed analysis tools. BY and S-HC contributed to the writing of the manuscript. All authors reviewed the manuscript.

### Conflict of Interest

The authors declare that the research was conducted in the absence of any commercial or financial relationships that could be construed as a potential conflict of interest.

## Supplementary Material

Supplementary Figure 1 Clustering dendrogram of samples to detect outliers.

Supplementary Figure 2 Clustering dendrogram of genes among CGGA mRNAseq_693. The colored row below the dendrogram indicates module membership identified by the dynamic tree cut method, and different colors represent different modules respectively.

Supplementary Figure 3 Venn diagram of the top 10 hub genes in brown module and the 13 genes of the MAPK signaling pathway. TNFRSF1A was the only common gene among the two cohorts.

Supplementary Table 1 Correlation of TNFRSF1A with clinicopathologic features of glioma patients in CGGA mRNA-array_301.

Supplementary Table 2 Correlation of TNFRSF1A with clinicopathologic features of glioma patients in CGGA mRNAseq_325.

Supplementary Table 3 Correlation of TNFRSF1A with clinicopathologic features of glioma patients in CGGA mRNAseq_693.

Supplementary Table 4 Correlation of TNFRSF1A with clinicopathologic features of glioma patients in GSE4271.

Supplementary Table 5 Correlation of TNFRSF1A with clinicopathologic features of LGG patients in TCGA.

Supplementary Table 6 Correlation of TNFRSF1A with clinicopathologic features of glioma patients in TCGA.
