# Supplementary figures and images for: Integrated Transcriptome Analyses and Experimental Verifications of Mesenchymal-Associated TNFRSF1A as a Diagnostic and Prognostic Biomarker in Gliomas
Source: Front Oncol. 2020 Mar 17;10:250. doi: 10.3389/fonc.2020.00250 (PMC7090130; doi:10.3389/fonc.2020.00250)

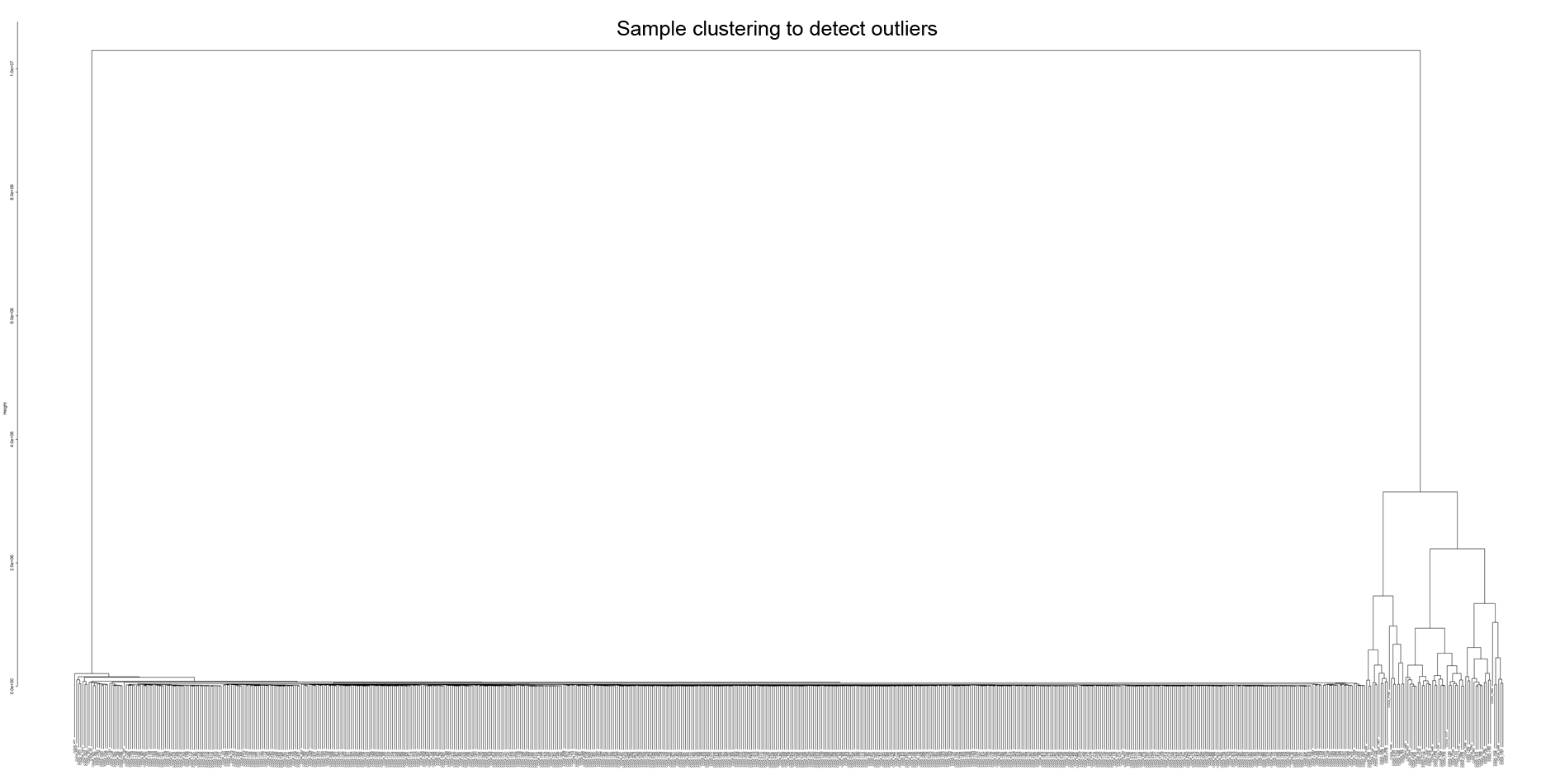

Supplement: Supplementary Figure 1 — Clustering dendrogram of samples to detect outliers. [file Image_1.tif]

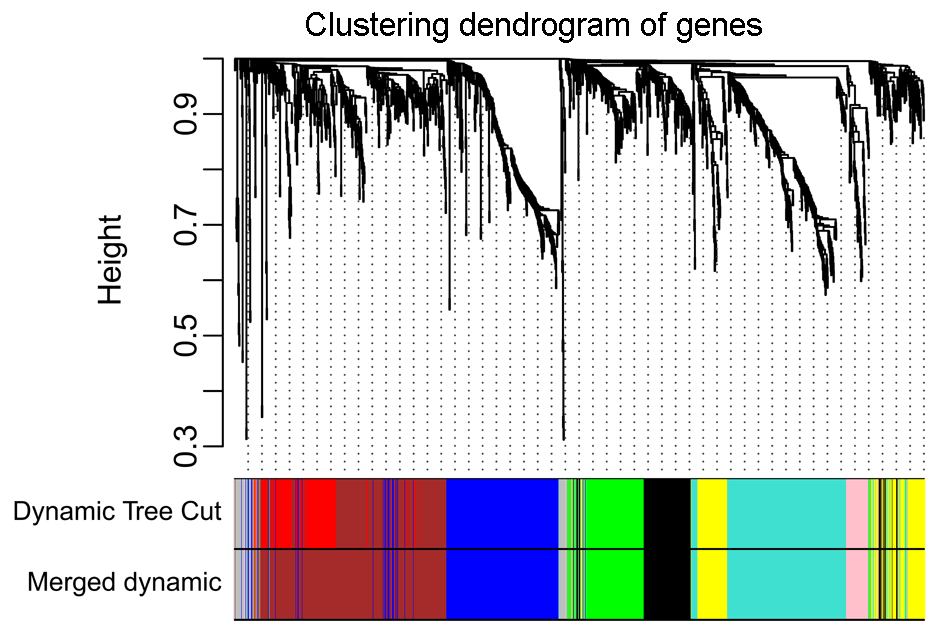

Supplement: Supplementary Figure 2 — Clustering dendrogram of genes among CGGA mRNAseq_693. The colored row below the dendrogram indicates module membership identified by the dynamic tree cut method, and different colors represent different modules respectively. [file Image_2.tif]

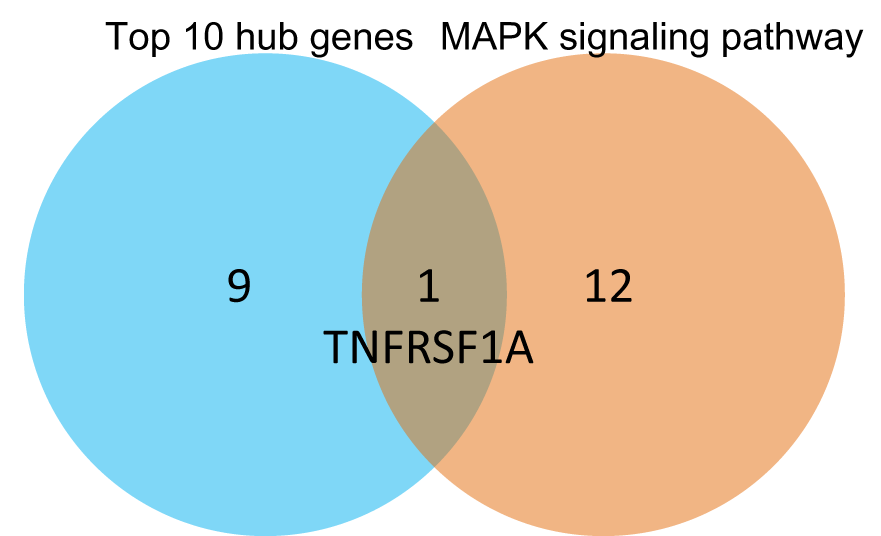

Supplement: Supplementary Figure 3 — Venn diagram of the top 10 hub genes in brown module and the 13 genes of the MAPK signaling pathway. TNFRSF1A was the only common gene among the two cohorts. [file Image_3.tif]
