# Supplementary material for: Integrated Transcriptome Analyses and Experimental Verifications of Mesenchymal-Associated TNFRSF1A as a Diagnostic and Prognostic Biomarker in Gliomas
Source: Front Oncol. 2020 Mar 17;10:250. doi: 10.3389/fonc.2020.00250 (PMC7090130; doi:10.3389/fonc.2020.00250)
Supplement: Supplementary Table 1 — Correlation of TNFRSF1A with clinicopathologic features of glioma patients in CGGA mRNA-array_301. [file Table_1.DOCX]

Supplementary Material

**Supplementary Table 1. Correlation of TNFRSF1A with clinicopathologic features of glioma patients in CGGA mRNA-array_301.**

| Variables | Case  (n=301) | TNFRSF1A | | P value |
| --- | --- | --- | --- | --- |
|  |  | Low (n=154) | High (n=147) |  |
| Age (years) |  |  |  | **0.001** |
| <60 | 269 | 145 | 124 |  |
| ≥60 | 30 | 7 | 23 |  |
| NA | 2 | 2 | 0 |  |
| Gender |  |  |  | 0.231 |
| Female | 121 | 67 | 54 |  |
| Male | 180 | 87 | 93 |  |
| WHO grade |  |  |  | **P<0.001** |
| Ⅱ | 117 | 85 | 32 |  |
| Ⅲ | 57 | 30 | 27 |  |
| Ⅳ | 124 | 38 | 86 |  |
| NA | 3 | 1 | 2 |  |
| Histology |  |  |  | **P<0.001** |
| A | 58 | 39 | 19 |  |
| AA | 12 | 4 | 8 |  |
| AO | 10 | 7 | 3 |  |
| AOA | 22 | 12 | 10 |  |
| GBM | 108 | 28 | 80 |  |
| O | 18 | 15 | 3 |  |
| OA | 36 | 30 | 6 |  |
| rA | 5 | 1 | 4 |  |
| rAA | 4 | 2 | 2 |  |
| rAO | 4 | 3 | 1 |  |
| rAOA | 5 | 2 | 3 |  |
| rGBM | 5 | 3 | 2 |  |
| sGBM | 11 | 7 | 4 |  |
| NA | 3 | 1 | 2 |  |
| PRS_type |  |  |  | 0.673 |
| Primary | 264 | 135 | 130 |  |
| Recurrent | 23 | 11 | 12 |  |
| Secondary | 11 | 7 | 4 |  |
| NA | 3 | 1 | 2 |  |
| TCGA_subtypes |  |  |  | **P<0.001** |
| Classical | 23 | 11 | 12 |  |
| Mesenchymal | 111 | 10 | 101 |  |
| Neural | 81 | 67 | 14 |  |
| Proneural | 86 | 66 | 20 |  |
| Radio_status |  |  |  | **0.023** |
| Negative | 38 | 26 | 12 |  |
| Positive | 249 | 121 | 128 |  |
| NA | 14 | 7 | 7 |  |
| Chemo_status |  |  |  | 0.107 |
| Negative | 126 | 69 | 57 |  |
| Positive | 151 | 68 | 83 |  |
| NA | 24 | 17 | 7 |  |
| IDH_mutation_status |  |  |  | **P<0.001** |
| Mutant | 134 | 94 | 40 |  |
| Wildtype | 165 | 59 | 106 |  |
| NA | 2 | 1 | 1 |  |
| 1p19q_Codeletion |  |  |  | **0.017** |
| Codel | 16 | 13 | 3 |  |
| Non-codel | 76 | 37 | 39 |  |
| NA | 209 | 104 | 104 |  |

Note: CGGA, the Chinese Glioma Genome Altas; WHO, World Health Organization; A, astrocytomas; AA, anaplastic astrocytomas; AO, anaplastic oligodendrogliomas; AOA, anaplastic oligoastrocytomas; GBM, glioblastoma multiforme; O, oligodendrogliomas; OA, oligoastrocytomas; rA, recurrent astrocytomas; rAA, recurrent anaplastic astrocytomas; rAO, recurrent anaplastic oligodendrogliomas; rAOA, recurrent anaplastic oligoastrocytomas; rGBM, recurrent glioblastoma multiforme; sGBM, secondary glioblastoma multiforme; TCGA, The Cancer Genome Atlas; NA, not analyze.

**Supplementary Table 2. Correlation of TNFRSF1A with clinicopathologic features of glioma patients in CGGA mRNAseq_325.**

| Variables | Case  (n=325) | TNFRSF1A | | P value |
| --- | --- | --- | --- | --- |
|  |  | Low (n=168) | High (n=157) |  |
| Age (years) |  |  |  | **P<0.001** |
| <60 | 292 | 161 | 131 |  |
| ≥60 | 33 | 7 | 26 |  |
| Gender |  |  |  | 0.258 |
| Female | 122 | 68 | 54 |  |
| Male | 203 | 100 | 103 |  |
| WHO grade |  |  |  | **P<0.001** |
| Ⅱ | 103 | 85 | 18 |  |
| Ⅲ | 79 | 48 | 31 |  |
| Ⅳ | 139 | 34 | 105 |  |
| NA | 4 | 1 | 3 |  |
| Histology |  |  |  | **P<0.001** |
| A | 33 | 26 | 7 |  |
| AA | 14 | 6 | 8 |  |
| AO | 9 | 9 | 0 |  |
| AOA | 27 | 16 | 11 |  |
| GBM | 85 | 14 | 71 |  |
| O | 26 | 25 | 1 |  |
| OA | 35 | 30 | 5 |  |
| rA | 6 | 2 | 4 |  |
| rAA | 14 | 4 | 10 |  |
| rAO | 3 | 2 | 1 |  |
| rAOA | 12 | 11 | 1 |  |
| rGBM | 24 | 8 | 16 |  |
| rOA | 3 | 2 | 1 |  |
| sGBM | 30 | 12 | 18 |  |
| NA | 4 | 1 | 3 |  |
| PRS_type |  |  |  | 0.197 |
| Primary | 229 | 126 | 103 |  |
| Recurrent | 62 | 29 | 33 |  |
| Secondary | 30 | 12 | 18 |  |
| NA | 4 | 1 | 3 |  |
| Radio_status |  |  |  | 0.492 |
| Negative | 51 | 24 | 27 |  |
| Positive | 258 | 135 | 123 |  |
| NA | 16 | 9 | 7 |  |
| Chemo_status |  |  |  | 0.093 |
| Negative | 124 | 70 | 54 |  |
| Positive | 178 | 83 | 95 |  |
| NA | 23 | 15 | 8 |  |
| IDH_mutation_status |  |  |  | **P<0.001** |
| Mutant | 175 | 129 | 46 |  |
| Wildtype | 149 | 38 | 111 |  |
| NA | 1 | 1 | 0 |  |
| 1p19q_Codeletion |  |  |  | **P<0.001** |
| Codel | 67 | 60 | 7 |  |
| Non-codel | 250 | 104 | 146 |  |
| NA | 8 | 4 | 4 |  |

Note: CGGA, the Chinese Glioma Genome Altas; WHO, World Health Organization; A, astrocytomas; AA, anaplastic astrocytomas; AO, anaplastic oligodendrogliomas; AOA, anaplastic oligoastrocytomas; GBM, glioblastoma multiforme; O, oligodendrogliomas; OA, oligoastrocytomas; rA, recurrent astrocytomas; rAA, recurrent anaplastic astrocytomas; rAO, recurrent anaplastic oligodendrogliomas; rAOA, recurrent anaplastic oligoastrocytomas; rGBM, recurrent glioblastoma multiforme; rOA, recurrent oligoastrocytomas; sGBM, secondary glioblastoma multiforme; NA, not analyze.

**Supplementary Table 3. Correlation of TNFRSF1A with clinicopathologic features of glioma patients in CGGA mRNAseq_693.**

| Variables | Case  (n=693) | TNFRSF1A | | P value |
| --- | --- | --- | --- | --- |
|  |  | Low (n=343) | High (n=350) |  |
| Age (years) |  |  |  | **0.001** |
| <60 | 609 | 315 | 294 |  |
| ≥60 | 83 | 27 | 56 |  |
| NA | 1 | 1 | 0 |  |
| Gender |  |  |  | 0.877 |
| Female | 295 | 145 | 150 |  |
| Male | 398 | 198 | 200 |  |
| WHO grade |  |  |  | **P<0.001** |
| Ⅱ | 188 | 123 | 65 |  |
| Ⅲ | 255 | 153 | 102 |  |
| Ⅳ | 249 | 67 | 182 |  |
| NA | 1 | 0 | 1 |  |
| Histology |  |  |  | **P<0.001** |
| A | 38 | 22 | 16 |  |
| AA | 34 | 17 | 17 |  |
| AO | 28 | 22 | 6 |  |
| AOA | 82 | 50 | 32 |  |
| GBM | 140 | 45 | 95 |  |
| O | 23 | 19 | 4 |  |
| OA | 77 | 54 | 23 |  |
| rA | 26 | 15 | 11 |  |
| rAA | 31 | 13 | 18 |  |
| rAO | 23 | 15 | 8 |  |
| rAOA | 57 | 36 | 21 |  |
| rGBM | 109 | 22 | 87 |  |
| rO | 7 | 3 | 4 |  |
| rOA | 17 | 10 | 7 |  |
| NA | 1 | 0 | 1 |  |
| PRS_type |  |  |  | **0.002** |
| Primary | 422 | 229 | 193 |  |
| Recurrent | 271 | 114 | 157 |  |
| Radio_status |  |  |  | 0.63 |
| Negative | 113 | 66 | 47 |  |
| Positive | 509 | 248 | 261 |  |
| NA | 71 | 29 | 42 |  |
| Chemo_status |  |  |  | **0.035** |
| Negative | 151 | 87 | 64 |  |
| Positive | 457 | 218 | 239 |  |
| NA | 85 | 38 | 47 |  |
| IDH_mutation_status |  |  |  | **P<0.001** |
| Mutant | 356 | 228 | 128 |  |
| Wildtype | 286 | 91 | 195 |  |
| NA | 51 | 24 | 27 |  |
| 1p19q_Codeletion |  |  |  | **P<0.001** |
| Codel | 145 | 103 | 42 |  |
| Non-codel | 478 | 173 | 305 |  |
| NA | 70 | 67 | 3 |  |

Note: CGGA, the Chinese Glioma Genome Altas; WHO, World Health Organization; A, astrocytomas; AA, anaplastic astrocytomas; AO, anaplastic oligodendrogliomas; AOA, anaplastic oligoastrocytomas; GBM, glioblastoma multiforme; O, oligodendrogliomas; OA, oligoastrocytomas; rA, recurrent astrocytomas; rAA, recurrent anaplastic astrocytomas; rAO, recurrent anaplastic oligodendrogliomas; rAOA, recurrent anaplastic oligoastrocytomas; rGBM, recurrent glioblastoma multiforme; rO, recurrent oligodendrogliomas; rOA, recurrent oligoastrocytomas; NA, not analyze.

**Supplementary Table 4. Correlation of TNFRSF1A with clinicopathologic features of glioma patients in GSE4271.**

| Variables | Case  (n=100) | TNFRSF1A | | P value |
| --- | --- | --- | --- | --- |
|  |  | Low (n=44) | High (n=56) |  |
| Age (years) |  |  |  | 0.193 |
| <60 | 69 | 34 | 35 |  |
| ≥60 | 8 | 2 | 6 |  |
| NA | 23 | 8 | 15 |  |
| Gender |  |  |  | **0.034** |
| Female | 32 | 19 | 13 |  |
| Male | 68 | 25 | 43 |  |
| WHO grade |  |  |  | **P<0.001** |
| Ⅲ | 24 | 20 | 4 |  |
| Ⅳ | 76 | 24 | 52 |  |
| Recurrence |  |  |  | 0.310 |
| Negative | 77 | 36 | 41 |  |
| Positive | 23 | 8 | 15 |  |
| Subtype |  |  |  | **P<0.001** |
| Mesenchymal | 35 | 1 | 34 |  |
| Proneural | 37 | 31 | 6 |  |
| Proliferative | 28 | 12 | 16 |  |
| Microvascular proliferation |  |  |  | **P<0.001** |
| Negative | 21 | 18 | 3 |  |
| Positive | 56 | 18 | 38 |  |
| NA | 23 | 8 | 15 |  |
| Necrosis |  |  |  | **P<0.001** |
| Negative | 27 | 21 | 6 |  |
| Positive | 50 | 15 | 35 |  |
| NA | 23 | 8 | 15 |  |

Note: WHO, World Health Organization; NA, not analyze.

**Supplementary Table 5. Correlation of TNFRSF1A with clinicopathologic features of LGG patients in TCGA.**

| Variables | Case  (n=529) | TNFRSF1A | | P value |
| --- | --- | --- | --- | --- |
|  |  | Low (n=265) | High (n=264) |  |
| Age (years) |  |  |  | **0.007** |
| <60 | 455 | 238 | 217 |  |
| ≥60 | 71 | 25 | 46 |  |
| NA | 3 | 2 | 1 |  |
| Gender |  |  |  | **0.032** |
| Female | 239 | 132 | 107 |  |
| Male | 288 | 132 | 156 |  |
| NA | 2 | 1 | 1 |  |
| Race |  |  |  | 0.711 |
| Asian | 8 | 5 | 3 |  |
| Black or african american | 22 | 10 | 12 |  |
| White | 486 | 244 | 242 |  |
| NA | 13 | 6 | 7 |  |
| WHO grade |  |  |  | **0.037** |
| Ⅱ | 223 | 123 | 100 |  |
| Ⅲ | 244 | 111 | 133 |  |
| NA | 62 | 31 | 31 |  |
| Histology |  |  |  | **P<0.001** |
| Astrocytoma, anaplastic | 129 | 38 | 91 |  |
| Astrocytoma, NOS | 66 | 25 | 41 |  |
| Mixed glioma | 132 | 64 | 68 |  |
| Oligodendroglioma, anaplastic | 82 | 56 | 26 |  |
| Oligodendroglioma, NOS | 118 | 81 | 37 |  |
| NA | 2 | 1 | 1 |  |
| KPS |  |  |  | 0.911 |
| <80 | 43 | 22 | 21 |  |
| ≥80 | 240 | 125 | 115 |  |
| NA | 246 | 118 | 128 |  |
| Treatment_or_therapy |  |  |  | **P<0.001** |
| Negative | 184 | 115 | 69 |  |
| Positive | 310 | 134 | 176 |  |
| NA | 35 | 16 | 19 |  |

Note: LGG, low-grade glioma; TCGA, The Cancer Genome Atlas; WHO, World Health Organization; KPS, Karnofsky performance score; NA, not analyze.

**Supplementary Table 6. Correlation of TNFRSF1A with clinicopathologic features of glioma patients in TCGA.**

| Variables | Case  (n=698) | TNFRSF1A | | P value |
| --- | --- | --- | --- | --- |
|  |  | Low (n=354) | High (n=344) |  |
| Age (years) |  |  |  | **P<0.001** |
| <60 | 535 | 313 | 222 |  |
| ≥60 | 159 | 39 | 120 |  |
| NA | 4 | 2 | 2 |  |
| Gender |  |  |  | 0.053 |
| Female | 298 | 164 | 134 |  |
| Male | 397 | 189 | 208 |  |
| NA | 3 | 1 | 2 |  |
| Race |  |  |  | 0.372 |
| Asian | 13 | 6 | 7 |  |
| Black or african american | 33 | 13 | 20 |  |
| White | 636 | 328 | 308 |  |
| NA | 16 | 7 | 9 |  |
| WHO grade |  |  |  | **P<0.001** |
| Ⅱ | 223 | 162 | 61 |  |
| Ⅲ | 244 | 138 | 106 |  |
| Ⅳ | 168 | 14 | 154 |  |
| NA | 63 | 40 | 23 |  |
| Histology |  |  |  | **P<0.001** |
| Astrocytoma, anaplastic | 129 | 50 | 79 |  |
| Astrocytoma, NOS | 66 | 40 | 26 |  |
| GBM | 168 | 14 | 154 |  |
| Mixed glioma | 132 | 90 | 42 |  |
| Oligodendroglioma, anaplastic | 82 | 64 | 18 |  |
| Oligodendroglioma, NOS | 118 | 95 | 23 |  |
| NA | 3 | 1 | 2 |  |
| TCGA_subtypes |  |  |  | **P<0.001** |
| Classical | 39 | 0 | 39 |  |
| Mesenchymal | 51 | 1 | 50 |  |
| Neural | 25 | 2 | 23 |  |
| Proneural | 38 | 11 | 27 |  |
| NA | 545 | 340 | 205 |  |
| KPS |  |  |  | 0.66 |
| <80 | 43 | 23 | 20 |  |
| ≥80 | 240 | 163 | 77 |  |
| NA | 415 | 168 | 247 |  |
| Treatment_or_therapy |  |  |  | **P<0.001** |
| Negative | 205 | 142 | 63 |  |
| Positive | 449 | 193 | 256 |  |
| NA | 44 | 19 | 25 |  |

Note: TCGA, The Cancer Genome Atlas; WHO, World Health Organization; GBM, glioblastoma multiforme; KPS, Karnofsky performance score; NA, not analyze.
